# The Role of DNA Mismatch Repair and Recombination in the Processing of DNA Alkylating Damage in Living Yeast Cells

**DOI:** 10.4236/abb.2015.66040

**Published:** 2015-06-01

**Authors:** Hernan Flores-Rozas, Lahcen Jaafar, Ling Xia

**Affiliations:** 1 College of Pharmacy and Pharmaceutical Sciences, Florida A&M University, Tallahassee, USA; 2 Institute of Molecular Medicine and Genetics, Georgia Regents University, Augusta, USA

**Keywords:** DNA Mismatch Repair, Recombination, DNA Damage, Non-Homologous End Joining

## Abstract

It is proposed that mismatch repair (MMR) mediates the cytotoxic effects of DNA damaging agents by exerting a futile repair pathway which leads to double strand breaks (DSBs). Previous reports indicate that the sensitivity of cells defective in homologous recombination (HR) to DNA alkylation is reduced by defects in MMR genes. We have assessed the contribution of different MMR genes to the processing of alkylation damage *in vivo*. We have directly visualized recombination complexes formed upon DNA damage using fluorescent protein (FP) fusions. We find that *msh*6 mutants are more resistant than wild type cells to MNNG, and that an *msh*6 mutation rescues the sensitivity of *rad*52 strains more efficiently than an *msh*3 mutation. Analysis of RAD52-GFP tagged strains indicate that MNNG increases repair foci formation, and that the inactivation of the *MHS*2 and MSH6 genes but not the MSH3 gene result in a reduction of the number of foci formed. In addition, in the absence of HR, NHEJ could process the MNNG-induced DSBs as indicated by the formation of NHEJ-GFP tagged foci. These data suggest that processing of the alkylation damage by MMR, mainly by MSH2-MSH6, is required for recruitment of recombination proteins to the damage site for repair.

## 1. Introduction

Mismatch repair (MMR) plays a crucial role in the maintenance of the genome by repairing nucleotide misincorporations that occur during DNA replication and by preventing recombination between diverged sequences [1] [2], for review see [3]. Defects in MMR genes lead to the accumulation of mutations and microsattelites instability [4] which is the underlying defect in Hereditary Non Polyposis Colon Cancer (HNPCC). To date, six MMR genes have been identified in eukaryotes: *MSH*2, *MSH*3 and *MSH*6, which are homologs of *E. coli* MutS, and *MLH*1, *MLH*3 and *PMS*2 (*PMS*1 in yeast) which are homologous to MutL [2]. The recognition step, which is the rate-limiting step of the reaction, is carried out by two heterodimers that possess differential specificity for mismatches. The MSH2-MSH6 complex repairs base-base mismatches and small loops, while the MSH2-MSH3 complex binds and repairs large loops [5]. Inactivation of MSH6 or MSH3 results in modest phenotype due to their redundancy, whereas inactivation of MSH2 completely impairs the recognition step. Besides their important role in correcting replication errors, MMR proteins have been recently reported to participate in cellular responses to some forms of DNA damage induced by certain anticancer drugs [5]. Among these are the SN1-type methylating agents like N-methyl-N'-nitro-N-nitrosoguanidine (MNNG). The O^6^-methylguanine (O^6^-meG) is a portion of the DNA damage caused by SN1 methylating drugs. The O6MeG DNA methyl-transferase (MTase) efficiently removes the methyl group [6] and puts back normal base pairing, preventing cells from mutagenesis and death [7]. However, in MTase-deficient bacterial, yeast and mammalian cells, the O^6^-meG persists and forms during replication, base pairs with both C and T generating *O*^6^-MeG:T or *O*^6^-MeG:C mismatches [8]. Cells deficient in the MMR proteins cannot recognize *O*^6^-MeG: T and are therefore highly resistant to toxic effects of methylating agents [9]–[11].

The mechanism by which MMR proteins mediate cytotoxic responses to MNNG has not yet been elucidated. More than one model exists to describe this effect. The most extensively studied model proposes that MMR mediates the cytotoxic effects of DNA damaging agents by exerting a futile repair pathway in which O^6^-meG continually produces a template for MMR system [12] and leads to repeated cycles of repair resulting in the formation of a single-strand gaps [13] opposite to O^6^-meG which finally results, during the following cycle of DNA replication, in double strand breaks (DSBs) which lead cell death if not repaired [14] [15]. The DSBs, caused by the collapse of replication forks, are in the most part repaired either by homologous recombination (HR) or nonhomologous end joining (NHEJ) [16]. However, the contribution of both repair pathways depends mostly on the cell-cycle stage, with NHEJ being used in G1 in which the two DSBsends are directly rejoined. On the other hand, HR is mainly active during S/G2 phases where sister chromatids are present [17]. The choice of the pathway differs also among species. In yeast, HR is the most dominant whereas, NHEJ is most used in higher eukaryotes [18]. In *S. cerevisiae*, HR is controlled by the *RAD*52 epistasis group which includes *RAD*50, *RAD*51, *RAD*52, *RAD*54, *RAD*55, *RAD*57, *RAD*59, *RDH*54/*TID*1, *MRE*11 and *XRS*2 genes [19] [20]. Among these, the *RAD*52 gene seems to be involved in all HR events including single-strand annealing and gene conversion [21]. When *RAD*52 is deleted, severe phenotype defects in recombination are manifested in these strains suggesting the fundamental role of this gene compared to other mutants of the same group [22]. *S. cerevisiae* also uses NHEJ to repair DSBs. The components of yeast NHEJ pathway are divided into three protein complexes [23] that include the exonuclease complex MRX (Mre11/Rad50/Xrs2) [24], the DNA end recognition protein complex yKu (YKU70/YKU80) which is initially recruited to the DNA damage site [25] and the DNA ligase (Dnl4/Lif1) [25] [26].

The goal of our study is to determine the role of the different MMR genes in the processing of DNA alkylating damage to DSBs and to visualize the recruitment of the recombination pathways during the repair process. Our data suggest that the MSH2-MSH6 and not the MSH2-MSH3 complex mediates the processing of alkylating DNAdamage to DSBs and that both NHEJ and HR recombination pathways are recruited to the DNA damage site for repair.

## 2. Materials and Methods

### 2.1. General Genetic Methods and Strains

Yeast extract/peptone/dextrose media, synthetic drop-out media were as described [27] [28]. Strains are derivative of HFY2001, a strain of S288c background (Table 1).

### 2.2. Strains

Gene-disrupted strains were constructed using a 3-step PCR method. We created DNA cassettes containing exogenic regions of the gene of interest (*i.e. RAD*52), flanking a selective marker (*i.e. HIS*3, *TRP*1, *URA*3). These disruption cassettes were integrated into the genomes by homologous recombination. Double knockout mutants were obtained by sequential gene disruption using different selective markers. All strains were confirmed by PCR. Strains expressing GFP-tagged proteins were constructed using a 3-step PCR method we created DNA fragments that contain the 5' end of the gene of interest (*i.e. MSH*2, *RAD*52) fused, in frame, to genes encoding fluorescent proteins (*i.e*. GFP, CFP, YFP). These PCR products were integrated into the genomes of either wild-type (RKY3023) or MMR-deficient cells (*i.e. msh*2, *msh*6 mutant) by homologous recombination, and the strains containing the proper fusion were selected and confirmed by PCR and sequencing. Strains containing two differently tagged genes were obtained by sequential transformation into the same strain.

### 2.3. Survival Assay

A stock solution of 1M MNNG (Sigma-Aldrich) was prepared in DMSO and stored in the dark at −20°C. Because of the estimated 45 min half-life of MNNG in aqueous solutions, all experiments were performed in liquid cultures as follows: The cells were inoculated from a YPD plate into 2 ml of liquid YPD medium and cultivated overnight. The cells were then diluted with YPD to OD_600_ ≈ 0.3, and 5 ml cultures were incubated for a further 3 – 4 hrs, when the cells were again in an exponential growth phase. The cells were then mock-treated and treated with several concentrations of freshly diluted MNNG for 1hr. They were harvested, washed, and spotted (~10 μl drops) at serial dilutions (1 × 10^−1^ – 10^−5^) on YPD plates. The plates were evaluated after 3 days of cultivation at 30°C. The results shown are based on 3 – 5 independent experiments.

### 2.4. Live Cell Imaging and Fluorescent Microscopy

The cells were prepared as described in spot assay. For visualizing DNA in living cells, 10 mg/ml DAPI was added to the culture 30 min before imaging. Next, 1 ml aliquots were washed, pelleted and resuspended in 200μl of fresh synthetic minimal medium (SD). Then 5 ml volume of cells were immobilized on a glass slide precoated with 0.1% poly-L-lysine solutions (Sigma), to prevent evaporation, the cover glass was sealed with Cytoseal™ XYL mounting medium (Richard-Allan Scientific). Cells were viewed under a Zeiss Axioplan 2 imaging microscope (Carl Zeiss, Thornwood, NY) with a water-immersion Achroplan 63_/0.9W/DIC III objective. The illumination source was a 100-W mercury arc lamp. Cell images were taken using an AxioCamHRm digital camera operated via AxioVision 4.5 software. Confocal images were captured with a LSM 510 META system operated via META 3.2 software. The wavelengths of the filters used to visualize the RAD52-GFP (excitation 488 nm; emission 509 nm) and 4',6-diamidino-2-phenylindole (DAPI; excitation 358 nm, emission 463 nm). Image acquisition times for RAD52-GFP was 1100 ms. At least 300 nuclei were counted for total of 6 – 10 field of cells, the observations of RAD52-GFP foci formation are from 1.5 to 6 hr after MNNG treatment, the results shown are based on 3 independent experiments. Unless otherwise noted, all experiments were performed at room temperature.

### 2.5. Immunofluorescence Staining

Yeast Cells were grown and treated with MNNG exactly as in the spot tests. Immunofluorescence staining was carried out as previously described [29] with some modifications: Paraformaldehyde was added to 2 – 5 ml cultures to a final concentration of 4% and incubated at room temperature for 1 hr with gentle agitation. Cells were washed five times with a solution containing 5 mM MgCl_2_, 40 mM KH_2_PO_4_ and resuspended in 0.5 ml of digestion buffer containing 5 mM MgCl_2_, 40 mM KH_2_PO_4_, and 1.2 M sorbitol. Zymolase (1 mg/ml final concentration) was added and cells were incubated at 30° for 45 – 60 min. After a wash with digestion buffer, cells were spotted on slides precoated with poly-L-lysine (Sigma-Aldrich) and incubated 15 – 30 min. Slides were then incubated in blocking buffer (1.5% BSA in PBS, 0.5% Tween 20, and 0.1% Triton X-100) for 30 min. Incubation with the primary antibody directed against H2A phosphoserine 129 (1:1000 dilution, gift from Dr. Bonner, NIH) was performed overnight at room temperature. The slides were then washed two to three times with PBS, once with blocking buffer and then incubated for 1 hr at room temperature with a secondary antibody conjugated with the green fluorescent dye Alexa 594 (Molecular probes, 1:500 dilution in blocking buffer). After three washes with PBS, slides were then stained with (10 μg/ml) of 4,6-diamino-2-phenylindole (DAPI), for 5 min at room temperature. The samples were then visualized under microscope.

### 2.6. Statistical Analysis

Data analysis and graphing was performed using the GraphPad Prism 4 software package. Specific analysis for each experiment is indicated in the figure legend. In most cases the mean of at least three experiments is plotted together with the standard deviation.

## 3. Results

### 3.1. Inactivation of MSH6 but Not MSH3 Rescues the Sensitivity of HR-Deficient Strains to MNNG

**Figure 1:**
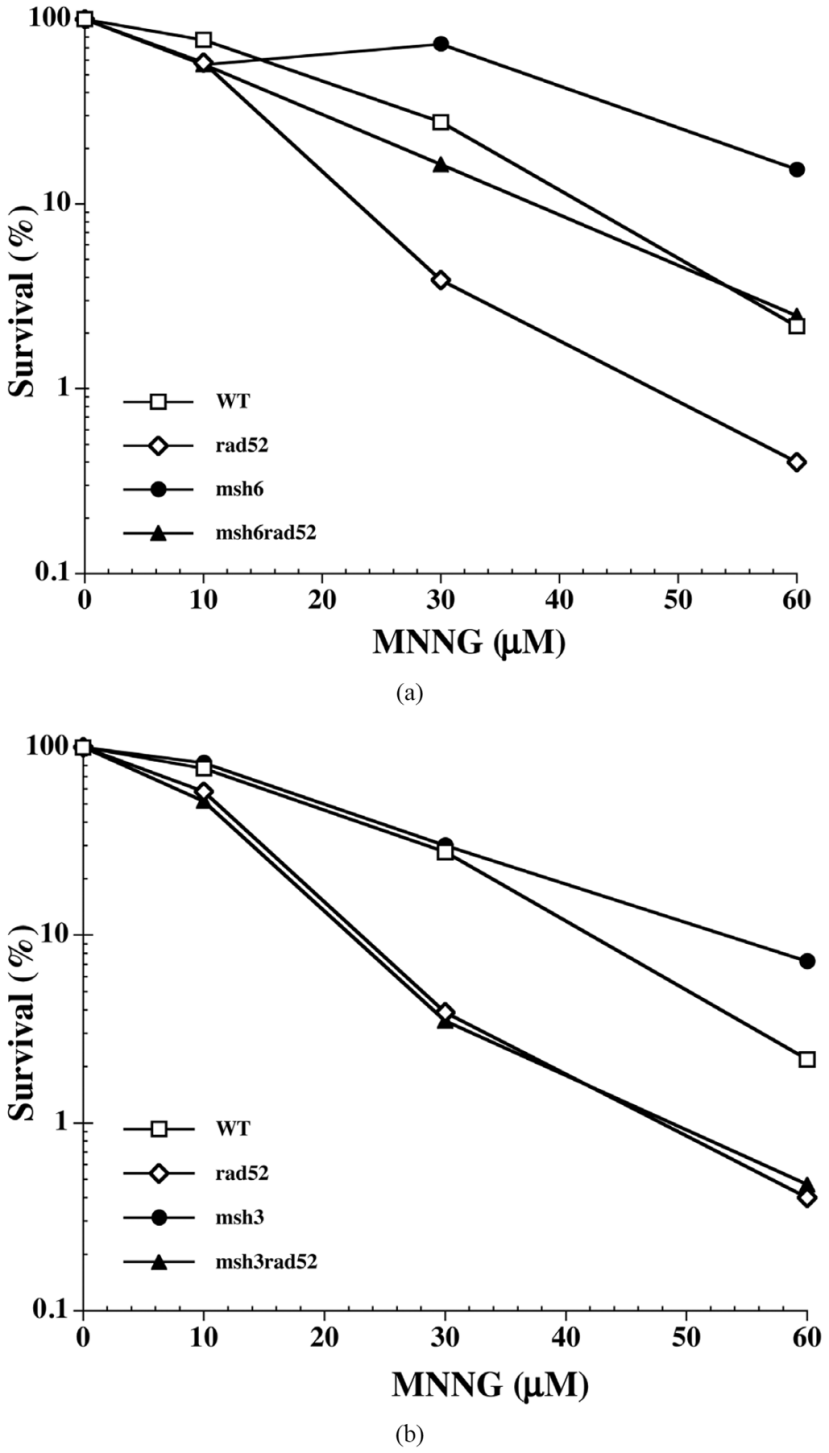
MNNG sensitivity of the MMR-deficient (*msh*3 or *msh*6) and/ or HR-deficient (*rad*52) strains. Mid-log phase cells were treated with the indicated concentrations of MNNG, harvested, washed and spotted onto YPD plates at proper serial dilutions as described under Materials and Methods. Each data point represents the mean of 3 independent experiments. (a) Sensitivity of *msh*6 and *msh*6-derived strains. The *msh*6 mutations increases the survival of cells to MNNG exposure and rescues *rad*52 mutants; (b) Sensitivity of *msh*3 and *msh*3-derived mutants to MNNG. Inactivation of *MSH*3 does not increase the survival of cells to MNNG, and does not rescue the sensitivity of the *rad*52 strain.

Exposure of yeast strains to MNNG resulted in significant loss of viability in a dose dependent manner. At 30 μM of MNNG, wild type cells displayed 40% viability, while *rad52* mutants were only 5% viable (Figure 1(a)). Interestingly, *msh*6 mutants were only slightly affected (viability of ~90%) at this concentration of the alkylating agent (Figure 1(a)). This is more apparent at 60 μM of MNNG where the viability of wild type cells was reduced to 4%, and that of *rad*52 to 0.5%, while the *msh*6 mutant was 30% viable (7-fold higher than wild type) (Figure 1(a)). However, inactivation of the *MSH*3 gene did not lead to an increase in the tolerance to alkylation damage, since the *msh*3 strain displays sensitivity to MNNG similar to that of wild type cells (Figure 1(b)). Inactivation of the *MSH*6 gene in a *rad*52 mutant results in increased resistance to MNNG treatment. The *msh*6 *rad*52 double mutant displays 35% survival at 30 μM MNNG, close to that observed in the wild type strain at this concentration (40% survival) and 7-fold higher than the *rad*52 strain, and at 60 μM MNNG it is identical to that of wild type cells (4%) and 8-fold higher than *rad*52 (Figure 1(a)). Conversely, inactivation of the *MSH*3 gene on the *rad*52 strain did not increase the survival to MNNG at any of the concentration tested (Figure 1(b)).

### 3.2. Exposure of Cells to MNNG Results in Formation of Repair Foci

**Figure 2:**
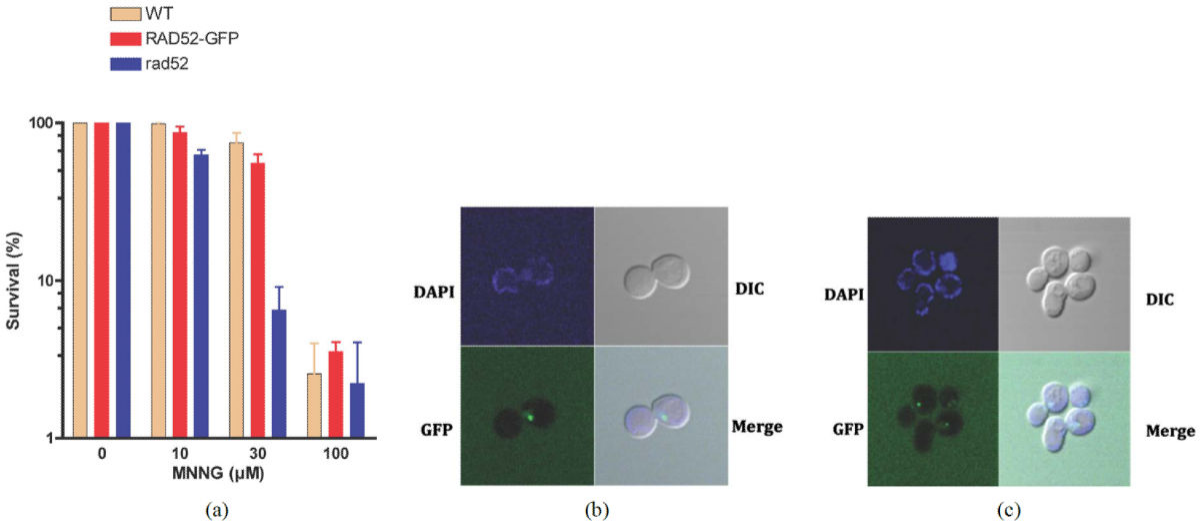
Formation of RAD52-GFP tagged strain upon DNA damage. (a) Survival of the *RAD*52 (WT), *RAD*52-*GFP* (WT) and *rad*52 (null) strains to MNNG exposure. Error bars show standard error of the mean from 3 independent experiments; (b) Visualization of RAD52-GFP foci upon exposure to γ-irradiation; (c) Visualization of RAD52-GFP foci upon exposure to MNNG.

To study the involvement of MMR genes in the processing of alkylation damage to DSBs, we constructed a series of strains that contain as a reporter RAD52 fused to a GFP at the C-terminal end. The RAD52-GFP strain behaves similarly to the wild type strain when exposed to DNA damaging agents that cause DSBs (Figure 2(a)) displaying a survival that is similar to that of wild type cells and significantly higher than that of the *rad*52 mutant, indicating that the GFP tag does not affect the activity of RAD52 and that the *RAD*52-*GFP* strain is competent in HR. The strain was tested for its ability to display repair foci after DNA damage. Treatment of the cells with ionizing radiation (γ-rays) resulted in a single defined foci, restricted to the nucleus, which appears within 30 min after exposure (Figure 2(b)) consistent with previous reports [30]. Similarly, exposure of the *RAD*52-*GFP* strain to MNNG also induced RAD52 foci formation (Figure 2(c)), indicating that MNNG generates a damage that serves as a substrate for the HR machinery. In both treatment procedures the majority of the cells (>98%) that display RAD52 foci present a single foci and very few present two or more foci (~2%) (data not shown).

### 3.3. Formation of RAD52 Foci upon Treatment with MNNG Requires the Activity MSH6 but Not MSH3

**Figure 3:**
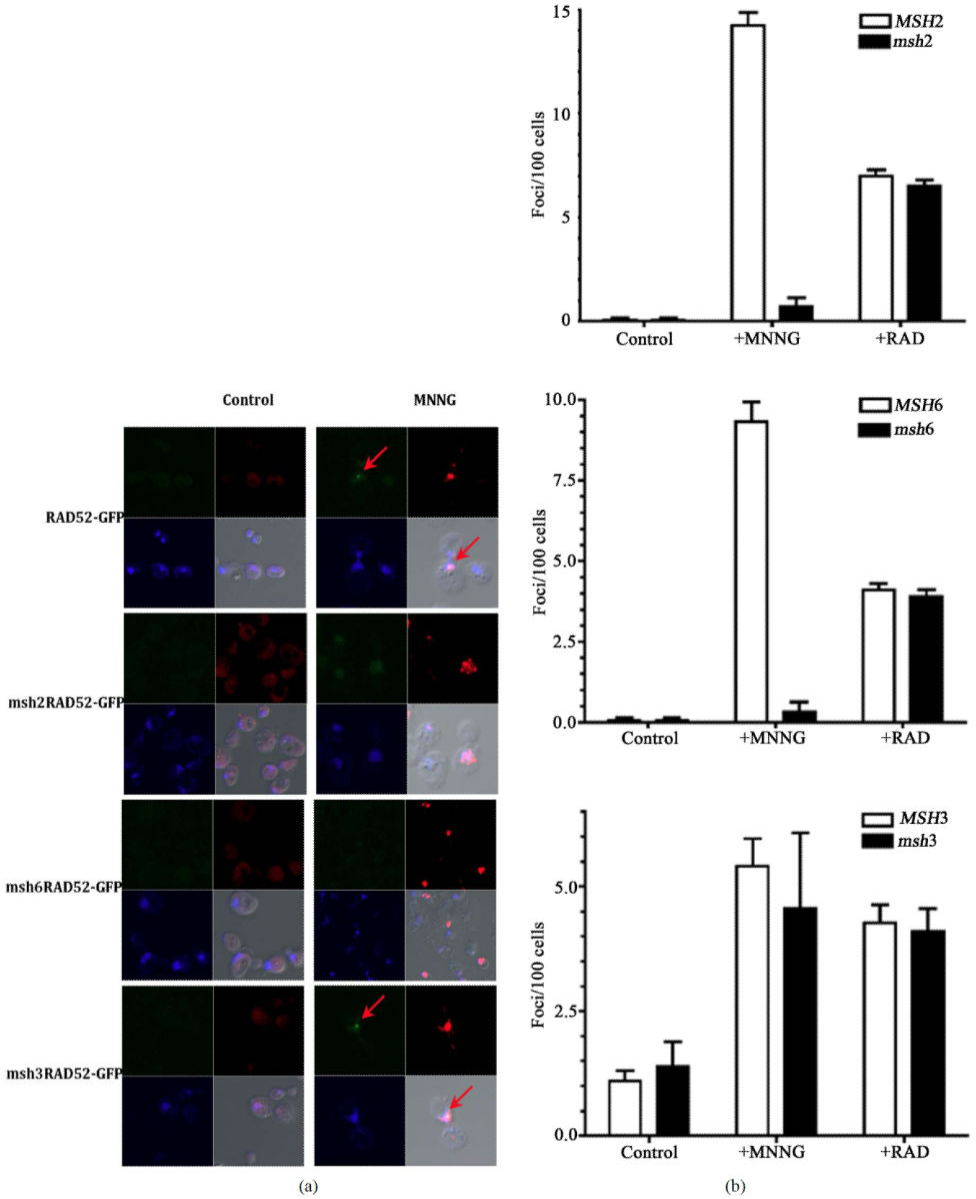
The MSH2-MSH6 complex is required for foci formation upon DNA damage. (a) Visualization of DNA damage sites as indicated by histone H2AX phosphorylation (red regions). MNNG treated or untreated (control) cells were fixed immediately after treatment with MNNG and processed for direct immunofluorescence microscopy using yeast γ-H2AX antibody as described in the Materials and Methods. Foci of Rad52-GFP (green spots) are indicated by an arrow. Nuclei were demarked by DAPI-stained (blue); (b) The numbers of RAD52-GFP foci formed was determined from 3 independent experiments visualizing at least 400 live yeast cells per experiment. Error bars indicate standard deviation. Strains used were wild type (*RAD*52-*GFP*), *msh*2 mutants (*msh*2 *RAD*52-*GFP*), *msh*3 mutants (*msh*3 *RAD*52-*GFP*) and *msh*6 mutants (*msh*6*RAD*52-*GFP*).

To determine if the recruitment of homologous recombination is altered in a MMR defective background, we inactivated MMR genes in the strain containing the RAD52-GFP fusion. Spontaneous foci form at very low frequency in the absence of exposure to the DNA alkylating agent (less than 1 per 100 cells, Figure 3(a) top left panel) and is increased at least 15-fold after treatment (Figure 3(a) top right panel and Figure 3(a)). Inactivation of *MSH*2 results in a considerable reduction of the RAD52 foci formed after exposure to MNNG (Figure 3(a)) to levels similar to those of unexposed cells (14-fold reduction, Figure 3(b)). Similarly, the inactivation of the *MSH*6 gene also reduces the accumulation of RAD52 foci (Figure 3(a)) by approximately 9-fold (Figure 3(b)). As expected, the inactivation of the *MSH3* gene did not significantly reduced the formation of RAD52 foci upon treatment with MNNG. As a control, exposure of the cells to ionizing radiation (+RAD) did not require a proficient MMR system since MMR mutants strains accumulate similar number of foci compared to the wild type strain (Figure 3(b)). In all cases that a RAD52 foci was observed, it was restricted to a specific damage area as indicated by histone γ-H2AX activation. Activation of γ-H2AX occurs predominantly after exposure to MNNG (Figure 3(a)). Interestingly, histone γ-H2AX still becomes activated in strains defective in MMR, which do not process the alkylation damage to DSB. This is consistent with observation that γ-H2AX activation is not restricted to DSBs but is a signal for DNA damage in general [31]. Consistent with the survival data (Figure 1(a) and Figure 1(b)), *MSH*2 and *MSH*6 genes, but not *MSH*3 gene, are required to process alkylation damage to an injury that requires HR for repair, most likely a DSB, as indicated by the formation of repair centers that contain RAD52.

### 3.4. Nonhomologous End Joining Can Be Recruited to DNA Damage Sites in the Absence of Homologous Recombination in a MMR Dependent Manner

**Figure 4:**
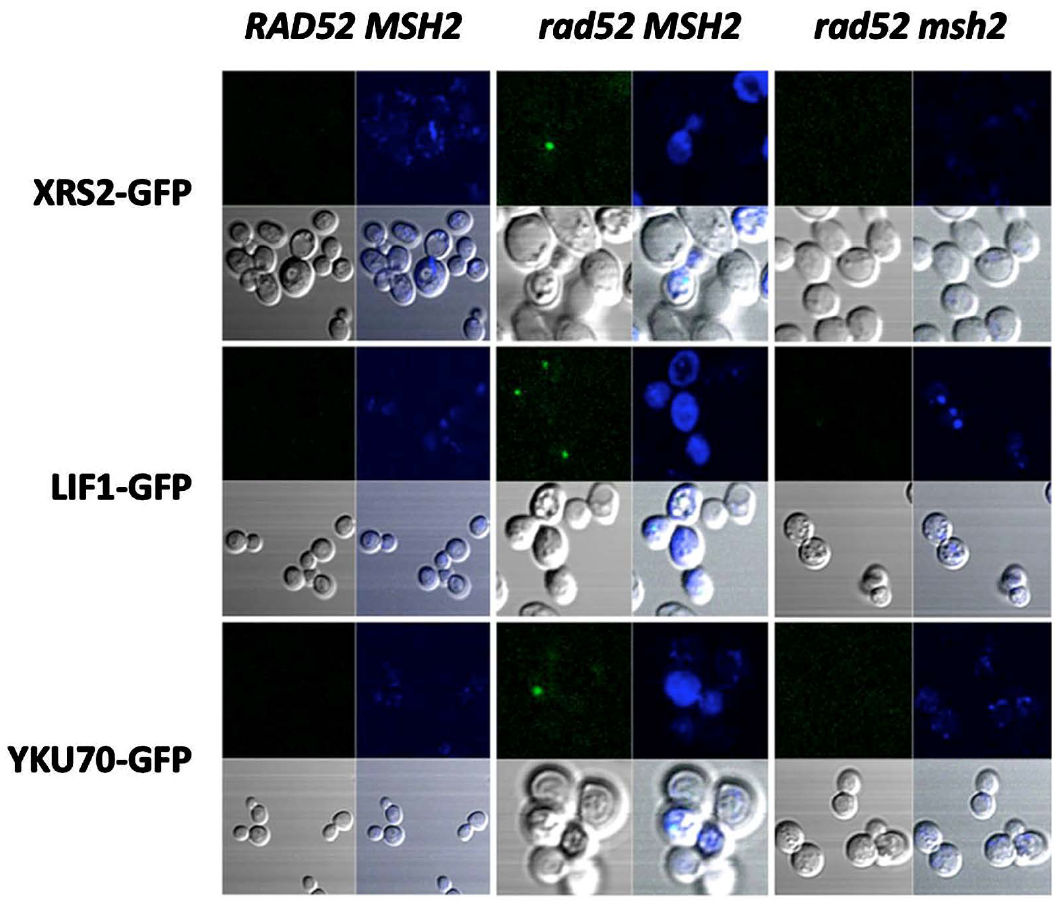
Inactivation of HR increases the frequency of MNNG-induced NHEJ foci dependent on mismatch repair. Stationary phase cells were treated with MNNG and immediately processed for live cell fluorescence microscopy as indicated in Materials and Methods. Visualization of NHEJ-GFP foci formation (green spots) in response to MNNG is presented. Nuclear DNA (blue) is stained with DAPI. In the HR proficient strains (*RAD*52 *MSH*2), no NHEJ foci were observed. When HR was inactivated (*rad*52 *MSH*2), distinct foci was observed in the *XRS*2-*GFP*, *LIF1-GFP* and *YKU*70-*GFP* strains. Foci formation was dependent on a functional MMR, since inactivation of *MSH*2 (*rad*52 *msh*2) significantly reduced the number of NHEJ foci observed, even in a HR deficient strain.

**Figure 5:**
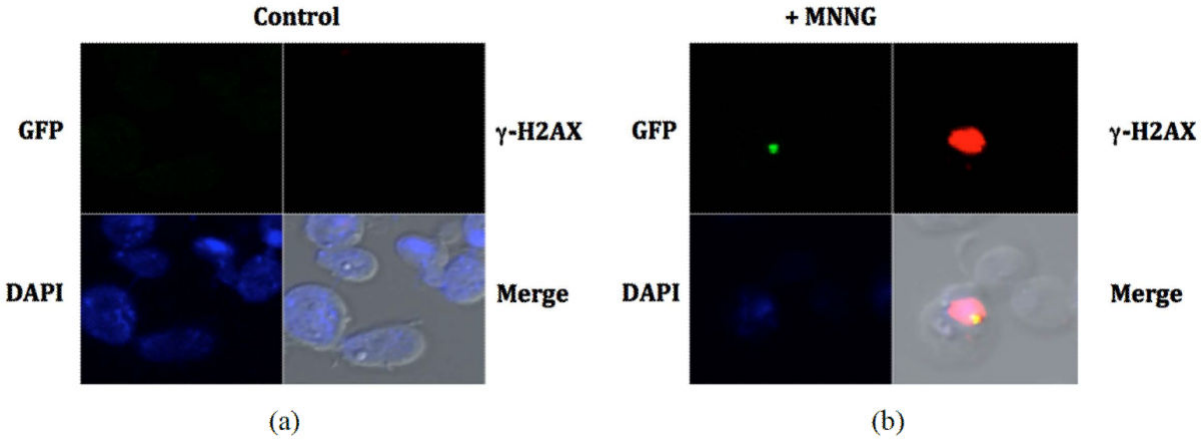
NHEJ repair foci assemble at damage sites in HR-defective strain. Upon exposure to MNNG, the *rad*52 *LIF1-GFP* strain displays repair foci (green spot) that localizes within DNA damage sites, as determined by activation of histone H2AX (red region). Cell nucleus was stained with DAPI (blue).

To determine if the repair of DNA damage resulting from the processing of DNA alkylation by MMR can also be repaired by NHEJ we constructed a series of strains containing GFP-tagged NHEJ proteins. Initial attempts to visualize DNA damage-induced foci of NHEJ proteins were unsuccessful. We reasoned that the presence of HR in the NHEJ-GFP tagged strains competes for DNA ends preventing the loading of NHEJ. We proceeded to inactivate HR in these strains and determine if foci form in a mismatch repair-dependent manner after exposure to MNNG. The strains defective in HR, which also contain fusions of a GFP at the C-terminus of YKu70, XRS2 and LIF1, appeared to be functional in DNA end joining as determined by their ability to repair a restricted plasmid compared to the NHEJ knock-out (data not shown). As shown in Figure 4, no NHEJ foci form in the presence of HR in the XRS2-GFP, LIF1-GFP and YKU70-GFP strains. However, upon inactivation of HR we observe NHEJ foci formation, although the frequency is much lower than what we observed with RAD52-GFP strains (only 3 - 5 fold over untreated cells). In addition, NHEJ foci formation after treatment with MNNG is also dependent on a proficient MMR pathway, since inactivation of *MSH*2 leads to a significant reduction of the observed foci. As in the RAD52 foci, the NHEJ foci is also restricted to sites of DNA damage as indicated by the colocalization of LIF1-GFP and activated histone γ-H2AX (Figure 5). These data suggests that NHEJ can also repair DNA damage that results from the processing of alkylated DNA by MMR.

## 4. Discussion

Defects in mismatch repair have been associated to increased tolerance to DNA damage. In particular, alkylation damage that results in methylation of O^6^-G can be processed to DSBs in a process that requires misincorporation by the DNA polymerase to form a T-methyl-O^6^-G that can be recognized by the MMR system. Attempts to repair this mismatch results in a futile cycle where MMR replaces the misincorporated T and DNA polymerase reintroduces it as long as the methyl-O^6^-G persists. It is speculated that this continuous cycle of repair eventually leads to DSBs which are toxic to the cell. We have investigated which component of MMR involved in the recognition step are required to process methylation damage to a DSB. We find that inactivation of *MSH*6, leads to an increased tolerance to MNNG, and that inactivation of *MSH*6 can rescue the sensitivity of the HR mutant *rad*52 to the alkylating agent. In contrast, inactivation of *MSH*3 does not alter the sensitivity of cells to DNA methylation and does not rescue the *rad*52 strain. This results are consistent with the fact that polymerase misincorporation opposite then methyl-O^6^-G leads to a base-base mispair, which are recognized by MSH2-MSH6 complex rather than the MSH2-MSH3 complex. A recent study that screened for mutants that display differential sensitivity to alkylation damage concluded that MMR is the only pathway that sensitizes cells to alkylation damage and that *MSH*3 has no involvement in this process [9], consistent with our results.

We have also shown that upon MNNG treatment, RAD52 foci form consistent with the involvement of HR in the repair of these lesions, and that the assembly of the repair center requires that MMR processes the DNA alkylation to a DSB. In fact, inactivation of *MSH*2 or *MSH*6 reduces the accumulation of RAD52 foci by 14- and 9-fold, respectively. On the contrary, inactivation of *MSH*3, did not affect the appearance of foci after treatment with MNNG. These results are consistent with our data on the sensitivity of the strains to exposure to the drug and suggest that the inability of recombination complexes to assemble and repair the damage results in cell death. We also determine if the alternative recombination pathway of non-homologous end joining could also be recruited to DNA damage ends. Strains that harbor GFP fusions of NHEJ components failed to accumulate foci upon treatment with MNNG unless HR has been inactivated. Although NHEJ foci were clearly visible, the frequency of foci formed was significantly lower than that observed for HR. This is consistent with the observation that in *S. cerevisiae* the main pathway for recombination is HR and that NHEJ plays a minor role in repair of DSB that may be restricted to G1 phase of the cell cycle. In fact the frequency of spontaneous NHEJ foci was at least 10 times lower (1 in 1000 cells) to that of RAD52 (1 in 100 cells) and upon DNA damage it only increased 3 - 5 fold. This suggests that even when inactivating HR, NHEJ does not become the default pathway, and that there may be additional mechanisms that control (limit) the recruitment of NHEJ complexes to DNA ends in addition to a potential competition by HR. Both repair foci formed (either HR or NHEJ) were limited to regions of DNA damage as indicated by activation of histone γ-H2AX. In eukaryotes, H2AX has been used extensively as s marker for DSBs. It is one of the earliest proteins to move to the DNA break where in yeast is phosphorylated in Ser-129 of the carboxy-terminal [32]. Interestingly, we observed that even in the absence of MSH2 or MSH6, H2AX becomes activated. However, no recruitment of RAD52 was visible, suggesting that a DSB has not been generated at that site. Although we cannot exclude other types of DNA damage (*i.e.* single DNA breaks, nicks, etc.) have been generated. It appears that activation of H2AX is not limited to generation of DSB, an observation that has been realized by others [31].

## Figures and Tables

**Table 1:** Yeast strains used in this study.

Strain	Genotype
HFY2001	Wild type
HFY2028	*msh*3::*hisGhis*
HFY2029	*msh*6::*hisGhis*
HFY2030	*mgt*1::*HIS*3
HFY2031	*rad*52::*HIS*3
HFY2032	*msh*3:: *hisGhis*, *rad*52::*URA*3
HFY2033	*msh*6::*hisGhis*, *rad*52::*HIS*3/*msh*6::*hisGhis*, *rad*52::*TRP*1
HFY2034	*RAD*52-*GFP*
HFY2035	*RAD*52-*GFP*, *msh*3::*TRP*1
HFY2036	*RAD*52-*GFP*, *msh*2::*TRP*1
HFY2037	*RAD*52-*GFP*, *msh*6::*URA*3
HFY2038	*KU*70-*GFP*
HFY2039	*KU*70-*GFP*, *rad*52::*URA*3
HFY2040	*KU*70-*GFP*, *msh*2::*kanMX*4
HFY2041	*KU*70*-GFP*, *rad*52::*URA*3*,msh*2::*kanMX*4
HFY2042	*LIF*1-*GFP*, *rad*52::*URA*3
HFY2043	*LIF*1-*GFP*, *msh*2::*kanMX*4
HFY2044	*LIF*1-*GFP*, *rad*52::*URA*3, *msh*2::*kanMX*4
HFY2045	*XRS*2-*GFP*, *rad*52::*URA*3
HFY2046	*XRS*2-*GFP*, *msh*2::*kanMX*4
HFY2047	*XRS*2-*GFP*, *rad*52::*URA*3*,msh*2::*kanMX*4

All strains are derivatives of HFY2001 (***MATa*** *his*3Δ200 *ura*3-52 *leu*2Δ1*trp*1Δ63 *ade*2Δ1 *ade*8 *hom*3-10*lys*2Δ*Bgl*), except for the NHEJ-GFP strains, which are derivatives of BY4741 (***MATa*** *his*3-1 *leu*2 *met*15*ura*3).
